# Sucrose is an early modulator of the key hormonal mechanisms controlling bud outgrowth in *Rosa hybrida*

**DOI:** 10.1093/jxb/erv047

**Published:** 2015-04-13

**Authors:** François Barbier, Thomas Péron, Marion Lecerf, Maria-Dolores Perez-Garcia, Quentin Barrière, Jakub Rolčík, Stéphanie Boutet-Mercey, Sylvie Citerne, Remi Lemoine, Benoît Porcheron, Hanaé Roman, Nathalie Leduc, José Le Gourrierec, Jessica Bertheloot, Soulaiman Sakr

**Affiliations:** ^1^Agrocampus-Ouest, Institut de Recherche en Horticulture et Semences (INRA, Agrocampus-Ouest, Université d’Angers), SFR 149 QUASAV, F-49045 Angers, France; ^2^INRA, Institut de Recherche en Horticulture et Semences (INRA, Agrocampus-Ouest, Université d’Angers), SFR 149 QUASAV, F-49071 Beaucouzé, France; ^3^Laboratory of Growth Regulators, Faculty of Science, Palacký University and Institute of Experimental Botany AS CR, Šlechtitelů 11, 78371 Olomouc, Czech Republic; ^4^Institut Jean-Pierre Bourgin, Unité Mixte de Recherche 1318, Institut National de la Recherche Agronomique–AgroParisTech, Institut National de la Recherche Agronomique Centre de Versailles-Grignon, 78026 Versailles cedex, France; ^5^UMR-CNRS-UP 6503, LACCO – Laboratoire de Catalyse en Chimie Organique, Equipe Physiologie Moléculaire du Transport de Sucres, Université de Poitiers, 40 av. du Recteur Pineau, 86022 Poitiers cedex, France; ^6^Université d’Angers, Institut de Recherche en Horticulture et Semences (INRA, Agrocampus-Ouest, Université d’Angers), SFR 149 QUASAV, F-49045 Angers, France

**Keywords:** Auxin, bud burst, cytokinins, *Rosa* sp., shoot branching, strigolactones, sugar, sugar signalling.

## Abstract

Sugar has only recently been identified as a key player in triggering bud outgrowth, while hormonal control of bud outgrowth is already well established. To get a better understanding of sugar control, the present study investigated how sugar availability modulates the hormonal network during bud outgrowth in *Rosa hybrida*. Other plant models, for which mutants are available, were used when necessary. Buds were grown *in vitro* to manipulate available sugars. The temporal patterns of the hormonal regulatory network were assessed in parallel with bud outgrowth dynamics. Sucrose determined bud entrance into sustained growth in a concentration-dependent manner. Sustained growth was accompanied by sustained auxin production in buds, and sustained auxin export in a *DR5::GUS*-expressing pea line. Several events occurred ahead of sucrose-stimulated bud outgrowth. Sucrose upregulated early auxin synthesis genes (*RhTAR1*, *RhYUC1*) and the auxin efflux carrier gene *RhPIN1*, and promoted PIN1 abundance at the plasma membrane in a *pPIN1::PIN1-GFP*-expressing tomato line. Sucrose downregulated both *RwMAX2*, involved in the strigolactone-transduction pathway, and *RhBRC1*, a repressor of branching, at an early stage. The presence of sucrose also increased stem cytokinin content, but sucrose-promoted bud outgrowth was not related to that pathway. In these processes, several non-metabolizable sucrose analogues induced sustained bud outgrowth in *R. hybrida*, *Pisum sativum*, and *Arabidopsis thaliana,* suggesting that sucrose was involved in a signalling pathway. In conclusion, we identified potential hormonal candidates for bud outgrowth control by sugar. They are central to future investigations aimed at disentangling the processes that underlie regulation of bud outgrowth by sugar.

## Highlight

Recent research shows that sugar availability triggers bud outgrowth. This paper further demonstrates that the effect of sucrose involves changes in the hormonal network related to bud outgrowth, and identifies potential hormones involved in sugar control.

## Introduction

Bud outgrowth is a highly controlled process by which the plant can adjust its development to environmental conditions. Among the internal regulators affecting this process, auxin (indole-3-acetic acid, IAA) produced by the young apical leaves was suggested to be responsible for the inhibition of lower axillary buds during apical dominance a long time ago (Thimann and Skoog, 1934; Cline, 1991; Domagalska and Leyser, 2011). Recently, less emphasis has been given to the role of auxin during apical dominance; rather, the involvement of sugar has been investigated. Indeed, after decapitation in pea, Morris *et al.* (2005) showed that initial bud outgrowth occurred prior to changes in auxin content in the adjacent stem tissues. Mason *et al.* (2014) support a theory in which loss of the shoot tip would remove a large sink for sugar and induce rapid distribution of sugar over long distances, which would be responsible for initial bud outgrowth. It is well established that bud outgrowth occurs along with a large induction of sugar metabolism and transport within buds (Marquat *et al.*, 1999; Decourteix *et al.*, 2008; Bonhomme *et al.*, 2010; Girault *et al.*, 2010; Henry *et al.*, 2011; Rabot *et al.*, 2012). Moreover, defoliation experiments (Mitchell, 1953; Kebrom *et al.*, 2010) and more recently the use of the wheat *tiller inhibition* mutant, in which sugars are diverted to highly elongating internodes (Kebrom *et al.*, 2012), suggest the control of bud outgrowth by sugar availability within the plant. Mason *et al.* (2014) demonstrated a causal relationship between sugar availability and bud outgrowth *in planta*: external sugar supply was sufficient to trigger bud outgrowth in non-decapitated pea plants. Similarly, a supply of sugar is necessary to trigger the outgrowth of buds *in vitro* (Henry *et al.*, 2011; Rabot *et al.*, 2012; Mason *et al.*, 2014). In this process, sucrose was suggested not only to play a trophic role, but also to act as a signalling entity, because palatinose, a non-metabolizable sucrose analogue, is able to trigger bud outgrowth (Rabot *et al.*, 2012). Although these findings highlight the importance of sugar as a trigger of bud outgrowth, the mechanisms underlying the induction of bud outgrowth by sugar are yet to be elucidated.

Much work has been done to unravel the inhibitory effect of auxin on bud outgrowth (Thimann and Skoog, 1934; Ferguson and Beveridge, 2009; Waldie *et al.*, 2010; Domagalska and Leyser, 2011). This effect is indirect, since apically derived auxin does not enter the buds (Hall and Hillman, 1975; Prasad *et al.*, 1993) and therefore two leading models, involving second messengers and the process of auxin canalization, have been proposed. The auxin canalization-based model is based on Sachs’ auxin transport model (Sachs, 1981; Bennett *et al.*, 2014). In this model, axillary buds are activated when the auxin initially flowing out of the bud is sufficient to trigger the establishment of a polar auxin transport canal connecting it to the auxin stream in the stem (Li and Bangerth, 1999; Domagalska and Leyser, 2011). The continual flow of auxin produced at the apex prevents axillary buds on the same axis from exporting their own auxin, thereby maintaining apical dominance. The establishment of polar auxin transport involves a regulatory positive feedback loop between the directional auxin flow and the polarization of the efflux facilitator PIN-FORMED proteins (PINs) at the plasma membrane in the direction of the initial flow (Bennett *et al.*, 2014). Strigolactones would act upstream of auxin by stimulating PIN removal from the plasma membrane, thus reducing the ability of the bud to create its own polar auxin transport (Shinohara *et al.*, 2013). In the second messenger-based model, auxin flow in the main stem negatively regulates the synthesis of cytokinins (Sachs and Thimann, 1967; Shimizu-Sato *et al.*, 2009) and positively regulates the levels of strigolactones (Brewer *et al.*, 2009), which act antagonistically on buds by inducing and inhibiting their outgrowth, respectively (Hayward *et al.*, 2009; Dun *et al.*, 2012). Within buds, the antagonistic effect of cytokinins and strigolactones is notably integrated by BRANCHED1 (BRC1), a transcription factor mainly expressed in non-growing axillary buds and with knock-out mutants that exhibit a highly branched phenotype (Aguilar-Martínez *et al.*, 2007; Braun *et al.*, 2012; Dun *et al.*, 2012).

In contexts other than bud outgrowth, several studies report that sugars control the biosynthesis, transport, or signalling of certain hormones, including auxin and cytokinins (Mishra *et al.*, 2009; LeClere *et al.*, 2010; Arrom and Munné-Bosch, 2012b; Sairanen *et al.*, 2012). In *Arabidopsis thaliana* roots, a genome-wide expression profiling study showed that glucose upregulated *YUCCA2*, an auxin biosynthesis gene, and two members of the auxin efflux gene family including *PIN1* (Mishra *et al.*, 2009). Moreover, sugars stimulated auxin biosynthesis by upregulating *ZmYUCC1* in developing maize kernels (LeClere *et al.*, 2010) or *AtTAA* and *AtYUCCA8* in *Arabidopsis* seedlings (Stewart Lilley *et al.*, 2012). Several genes involved in cytokinin metabolism are also regulated by sugars in *Arabidopsis* (Kushwah and Laxmi, 2013). Glucose upregulates the cytokinin biosynthesis gene *IPT3*, and has a differential effect on the expression of cytokinin catabolism-related genes: it upregulates *CKX4* and downregulates *CKX5*. Unlike for auxin and cytokinins, no report is currently available about the relationships between sugars and strigolactones, although we know that their biosynthesis and signalling pathways are under the control of phosphate and nitrogen (Czarnecki *et al.*, 2013; Sun *et al.*, 2014).

The objective of the present study was to determine whether sucrose and its signalling component modulate early hormonal homeostasis during bud outgrowth in *Rosa hybrida*. To monitor sugar availability for buds, we cultivated bud-bearing stem segments *in vitro* and supplied them with different sugar conditions, including different sucrose concentrations and non-metabolizable sucrose analogues (Chatfield *et al.*, 2000; Henry *et al.*, 2011; Rabot *et al.*, 2012). To assess the sequence of events, we monitored the temporal patterns of bud outgrowth and hormonal state. We used several techniques to characterize hormonal state, including the determination of hormone levels and gene expression, and imaging of reporter genes. This latter technique required the use of specific mutants of other species (tomato and pea). We thus demonstrated that sucrose availability modulates the entrance of buds into sustained growth, and that this effect is conserved across species. This impact is preceded by an early modification of hormonal homeostasis. We also identified some components of the hormonal network as potential candidates explaining the regulation of bud outgrowth by sucrose.

## Materials and methods

### Plant culture, *in vitro* cultivation of axillary buds and growth analysis

For the experiments on *R. hybrida* L. ‘Radrazz’, cuttings from cloned mother plants were grown in a greenhouse where the temperature was maintained around 22°C. Extra light was supplied by high-pressure sodium vapour lamps below 200W m^–2^. Water and mineral nutrients were provided by sub-irrigation for 10min day^–1^. Nodes from the median part of the stem were harvested on single-axis plants when the floral bud was visible, as previously described (Girault *et al.*, 2010).

For the experiment on *A. thaliana* (L.) Heynh., wild-type (WT) Columbia-0 was used. Seeds were sown and stratified for 48h at 7°C, then plants were grown in a growth chamber with a 16h day length at a temperature of 20/18°C (day/night). After 6 weeks of culture, first and second nodes bearing 1-mm-long buds were harvested on secondary flowering branches.

For the experiments on *Pisum sativum* L., the W6 22593 genotype was used for the WT, and DR5::GUS DSB2024, containing an auxin-inducible promoter fused with the β-glucuronidase reporter (DeMason and Polowick, 2009), was used to visualize auxin export. Plants were sown and grown in the growth chamber in the same conditions as for the *Arabidopsis* experiments, except stratification, which was not performed on this species. The third basal leaf-bearing node of single-axis plants was harvested when the fourth leaf was totally expanded.

For the experiment on *Solanum lycopersicum* L., the ‘Money Maker’ genotype was used as the WT and the *pAtPIN1::AtPIN1-GFP*-expressing line (Bayer *et al.*, 2009) was used to visualize PIN1 localization within bud stem. Plants were sown and grown in the growth chamber in the same conditions as for the *Arabidopsis* experiments, except stratification, which was not performed on this species. The second basal leaf-bearing node of single-axis plants was harvested when the plants were about 15cm long (3 to 4-week-old plants).

Once harvested, 1.5-cm stem segments were grown *in vitro* on classical solid MS medium (Duchefa) (1% gelose, aubygel) supplemented with different sucrose concentrations or different non-metabolizable analogues (palatinose, glucose[1→6]fructose; turanose, glucose[1→3]fructose; melibiose, galactose[1→6]glucose; and lactulose, galactose[1→4]fructose). These sugar analogues were initially used at 80mM for rose (Loreti *et al.*, 2000) and then at 30mM for herbaceous plants (pea and *Arabidopsis*), which was sufficient to induce bud outgrowth. In all experiments, mannitol was used as an osmotic control, as it is not metabolized by *R. hybrida* (Henry *et al.*, 2011) and is non-toxic for bud outgrowth (Supplementary Figure S1). *In vitro* excised buds were grown in a growth chamber (Strader) with a 16-h day length at a temperature of 23/20°C (day/night).

For the work shown in Fig. 3, buds were treated with 1-*N*-naphthylphthalamic acid (NPA), an auxin transport inhibitor, by deposition, on a bud, of a drop of unsolidified mixture containing 1% gelose, 1% PEG, 0.01% Tween-20, and 0.2% DMSO supplemented with 1mM NPA. The control corresponded to the same mixture without NPA.

For the work shown in Fig. 6, synthetic cytokinin 6-benzylaminopruine (BAP) and inhibitors of cytokinin synthesis (lovastatine; Hartig and Beck, 2005) and signalling (LGR-991 and PI-55; Nisler *et al.*, 2010), were added in the growth medium at 10 µM.

Once *in vitro*, buds were imaged daily and their elongation was quantified as described in the Supplementary material and in Supplementary Figure S2.

### Free IAA content analysis

For each sample, 5mg of frozen tissue was extracted with 50mM phosphate buffer containing an internal standard of [^2^H_5_] IAA at a concentration of 100fmol mg^–1^ of plant material. The extract was subjected to solid-phase extraction as described previously (Pencík *et al.*, 2009) and free IAA was analysed three times by ultra-high performance chromatography coupled with tandem mass spectrometry.

### Cytokinin content analysis

For each sample, 10mg of freeze-dried powder was extracted with 0.8ml of acetone/water/acetic acid (80/19/1, v/v/v), and 10 stable-labelled isotopes (OLChemIm) were used as internal standards and added as follows: 1ng of ^2^H_5_-t-Z7G (*trans*-zeatin-7-glucoside), 1ng of ^2^H_5_-t-Z9G (*trans*-zeatin-9-glucoside), 1ng of ^2^H_5_-t-ZOG (*trans*-zeatin *O*-glucoside), 1ng of ^15^N-t-Z (*trans*-zeatin), 1ng of ^2^H_5_-t-ZROG (*trans*-zeatin riboside *O*-glucoside), 1ng of ^2^H_5_-t-ZR (*trans*-zeatin riboside), 1ng of ^2^H_6_-iPRMP (isopentenyl adenosine monophosphate), 1ng of ^2^H_6_-iP (isopentenyl adenine), 1ng of ^2^H_5_-t-ZRMP (*trans*-zeatin riboside monophosphate), 0.1ng of ^15^N-iPR (isopentenyl adenosine). The extract was vigorously shaken for 1min, sonicated for 1min at 25 Hz, shaken for 10min at 4°C in a Thermomixer (Eppendorf), and then centrifuged (8000*g*, 4°C, 10min). The supernatants were collected, and the pellets were re-extracted twice with 0.4ml of the same extraction solution, then vigorously shaken (1min) and sonicated (1min, 25 Hz). After centrifugation, the three supernatants were pooled and dried (final volume 1.6ml).

Each dry extract was dissolved in 140 µl of acetonitrile/water (50/50, v/v), filtered, and analysed using a Waters Acquity ultra performance liquid chromatograph coupled to a Waters Xevo Triple quadrupole mass spectrometer TQD (UPLC-ESI-MS/MS). The compounds were separated on a reverse-phase column (Uptisphere C18 UP3HDO, 100×2.1mm × 3 µm particle size; Interchim, France) using a flow rate of 0.4ml min^–1^ and a binary gradient: (i) acetic acid, 0.1% in water (v/v); and (ii) acetonitrile, with 0.1% acetic acid. The solvent gradient was applied as follows [*t* (min), % A]: (0, 95%), (12, 40%), (13, 0%), (16, 95%); the column temperature was 40°C. Mass spectrometry was conducted in electrospray and Multiple Reaction Monitoring (MRM) scanning mode, in negative ion mode. Relevant instrumental parameters were set as follows: capillary 1.5kV (negative mode); source block and desolvation gas temperatures 130 and 500°C, respectively. Nitrogen was used to assist the cone and desolvation (150 and 800 l h^–1^, respectively); argon was used as the collision gas at a flow of 0.18ml min^–1^. The parameters used for MRM quantification of the different hormones are shown in Supplementary Table S1A.

The amounts of ZOG and ZROG were expressed as a ratio of standard peak areas (^2^H_5_-t-ZOG 287 > 225 and ^2^H_5_-t-ZROG 519 > 225) per unit dry weight, due to impurities contained in the samples. These matrix impurities co-eluted with the ZROG or ZOG peak.

Samples were reconstituted in 140 µl of 50/50 acetonitrile/H_2_O (v/v) per ml of injected volume. The limit of detection (LOD) and limit of quantification (LOQ) were extrapolated for each hormone from calibration curves and samples using the Quantify module of MassLynx software, version 4.1. The LODs and LOQs are listed in Supplementary Table S1B.

### Gene molecular cloning

5′ and 3′ cDNA ends were amplified using GeneRacer technology (Life Technologies), and then full-length cDNAs of each gene were cloned. The amplified fragments were cloned into the pGEM-T-easy vector (Promega) and transfected into *Escherichia coli* JM109 (Promega). Plasmids were purified using NucleoSpin Plasmid mini prep (Macherey-Nagel) and sequenced (GATC Biotech, Germany). Gene identity was determined based on their homology with *Arabidopsis* gene sequences and the presence of putative conserved domains in the corresponding peptide sequence (Supplementary Table S2). *RwMAX1/2/3/4* and *RhPP2A* were identified in previous studies (Klie and Debener, 2011; Djennane *et al.*, 2014)

### Quantification of gene expression

Total RNAs from ground frozen samples were extracted from buds using an RNA NucleoSpin kit (Macherey-Nagel). Genomic DNA was removed by incubating RNAs with DNase (Biolabs) for 10min at 37°C (1 µl of DNase for 10 µg of RNA). The reaction was stopped by adding EDTA at a final concentration of 5mM followed by 10min at 75°C. The absence of contamination by genomic DNA was checked by PCR using a specific primer designed against an intron region of the *RhGAPDH* gene (Girault *et al.*, 2010; Henry *et al.*, 2011). cDNAs were obtained by reverse transcription performed on 1 µg of RNA using SuperScript III Reverse Transcriptase (Invitrogen). Quantitative real-time PCR (qRT-PCR) was performed with SYBR Green Supermix (Biorad) using cDNA as a template, with the following programme: 2min at 50°C, 10min at 95°C, then 40 cycles of 15 s at 95°C and 60 s at 60 °C. The primers used for the qRT-PCR are given in Supplementary Table S3. Specific sets of primers were selected according to their melting curves. Fluorescence detection was performed using a Chromo4 Real-time PCR detector (Biorad). Quantification of relative gene expression was determined using *RhSAND1* expression as an internal control (Henry *et al.*, 2011; Rabot *et al.*, 2012).

### Confocal laser-scanning microscopy and GFP quantification

Longitudinal hand sections of *in vitro*-cultivated bud-bearing stem segments of *pAtPIN1::AtPIN1-GFP*-expressing tomato line were observed using a confocal laser scanning microscope (NIKON Eclipe Ti) with a 20× water immersion objective (excitation wavelength 488nm, emission spectra between 500 and 550nm). Laser power remained unchanged throughout the experiment. Quantification of the GFP signal was performed on 2D images using ImageJ software. Representative micrographs are given in Supplementary Figure S4. Integrated density of grey was determined on the 10 most intensely polarized plasma membrane poles of cells for each sample. The results are the means of three to four for replicates for each condition.

### GUS staining and light microscopy

Bud-bearing stem segments of garden pea were harvested in 85% (v/v) ice-cold acetone, rinsed in distilled water, infiltrated with a GUS-staining solution (Na_2_HPO_4_, 68mM; NaH_2_PO_4_, 32mM; 0.2% Triton X-100; potassium ferrocyanide, 0.5mM; potassium ferricyanide, 0.5mM; and 0.5mg ml^–1^ of 5-bromo-4-chloro-3-indolyl-D-glucuronic acid) under vacuum and incubated at 37°C overnight. The samples were then cleared with ethanol:ethyl acetate 3:1 (v:v) prepared one week before and observed under a binocular microscope (Leica).

### Statistical analyses

Statistical analyses were done using the Rcmdr package of R software for Windows (R Development Core Team, 2011). One-way ANOVA (α = 0.05) was run to test for the effects of sugar conditions on bud outgrowth. Significant differences are indicated by different letters or asterisks directly on the figures.

## Results

### Impact of sucrose on bud outgrowth dynamics

**Fig. 1.**
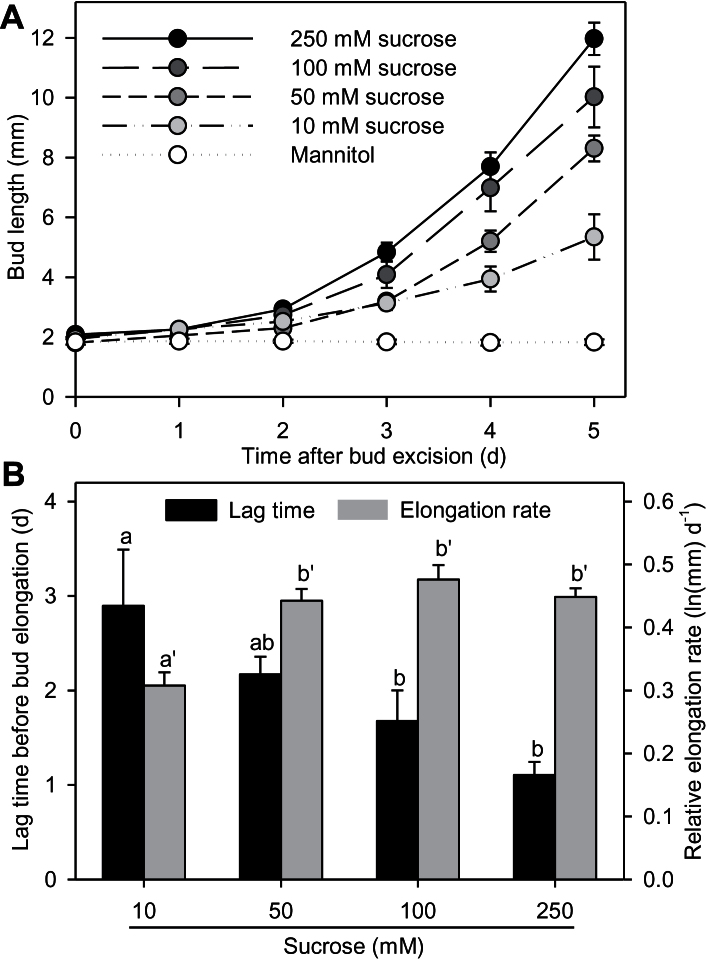
(A) Bud elongation. (B) Estimated lag times before elongation and relative elongation rates of buds cultivated *in vitro.* Experiments were conducted with 100mM mannitol or 10, 50, 100, or 250mM sucrose. Data are mean ± SE of 10 replicates. Letters indicate significant differences between means.

*In vitro* cultivated buds elongated within the first 6 days with all the sucrose concentrations tested, whereas no sustained bud outgrowth was observed in the presence of mannitol, used as an osmotic control (Fig. 1A). Initial slow growth was detected visually with mannitol, just after stem excision from the plant (data not shown), but could not be quantified from bud pictures. Sucrose stimulated bud elongation in a concentration-dependent manner. After 5 days of incubation, the average length of the buds kept on 10mM sucrose was 5.3mm, but was more than doubled (12.0mm) on 250mM sucrose. With sucrose, bud outgrowth showed a first phase of slow elongation, followed by a phase of rapid elongation. Fitting a two-phase exponential function on bud elongation (Supplementary Figure S2) revealed that increasing sucrose concentration gradually shortened the duration before the phase of rapid growth (2.9 and 1.1 days with 10 and 250mM sucrose, respectively; Fig. 1B). Moreover, at 10mM, the elongation rate of the rapid growth phase was reduced [(0.31 ln(mm) day^−1^] compared to higher concentrations [about 0.45 ln(mm) day^−1^], but there was no significant difference between concentrations above 50mM. These results demonstrate that sucrose is necessary for the transition between slow and rapid growth and modulates the timing of the transition in a concentration-dependent manner.

### Impact of non-metabolizable sucrose analogues on bud outgrowth

**Fig. 2.**
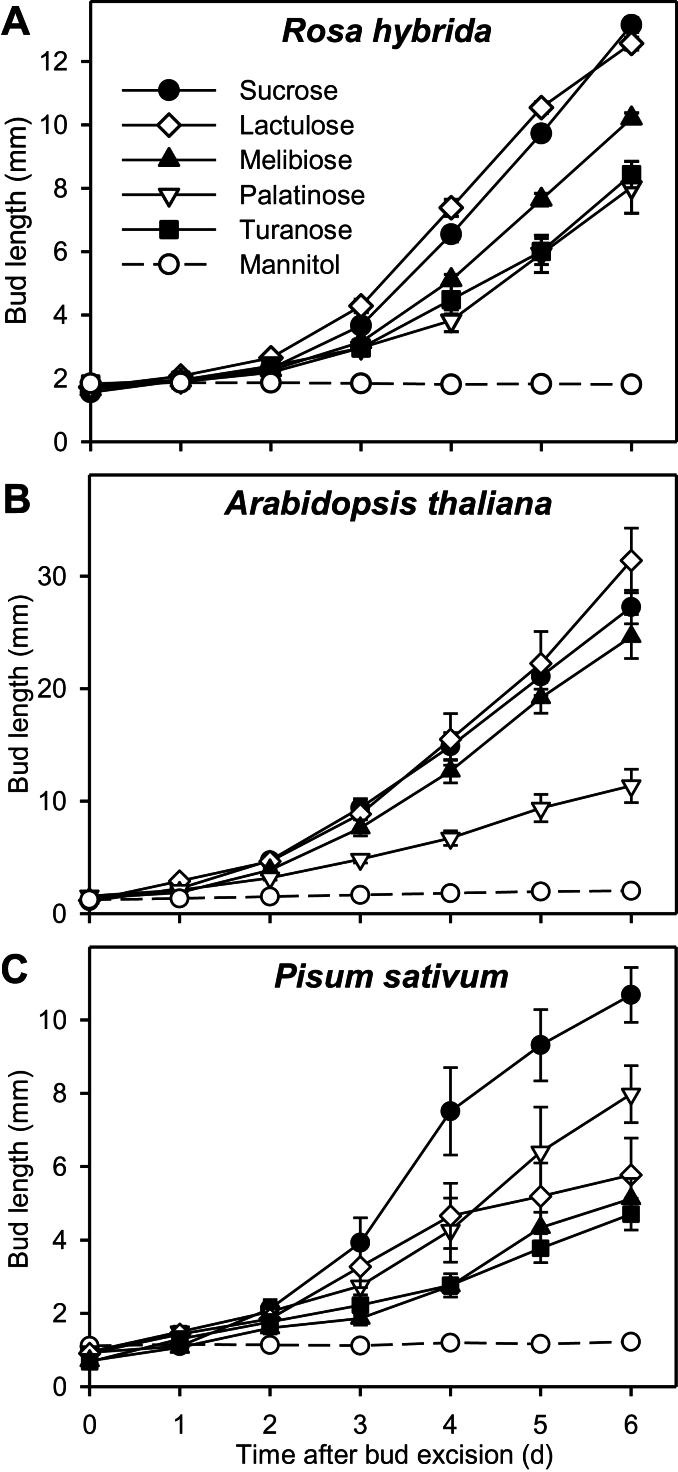
Elongation of buds grown with (A) 80mM mannitol, sucrose or non-metabolizable sucrose analogues (lactulose, melibiose, turanose, and palatinose) for *R. hybrida*, or with 30mM sucrose or 30mM non-metabolizable sucrose analogues for (B) *Arabidopsis* and (C) *P. sativum*. Data are mean ± SE of 8–10 replicates.

In order to confirm the previously suggested signalling role played by sucrose during bud outgrowth (Rabot *et al.*, 2012), the effect of several non-metabolizable sucrose analogues was investigated on *in vitro*-cultivated buds. In *R. hybrida*, buds grew out with all sucrose analogues (Fig. 2A). Lactulose yielded the same bud elongation pattern as sucrose (13mm after 6 days). In contrast, after 6 days, buds were shorter with melibiose (10mm), palatinose, and turanose (8mm) than with sucrose or lactulose.

Similarly, buds of *Arabidopsis* and *P. sativum* also grew out in the presence of sucrose or sucrose analogues but not in presence of mannitol (Fig. 2B, C). In *Arabidopsis*, palatinose triggered weaker bud elongation (11.4mm after 6 days) than sucrose, lactulose, and melibiose (27, 31, and 25mm, respectively, after 6 days). In pea, all the sucrose analogues had a weaker effect than sucrose (6, 8, and 5mm with melibiose, palatinose, and turanose, respectively, compared to 11mm with sucrose, after 6 days). These data suggest that sucrose can play a signalling role in the processes governing bud entrance into rapid growth and that this effect is conserved across species.

### Impact of sucrose on auxin export from buds.

**Fig. 3.**
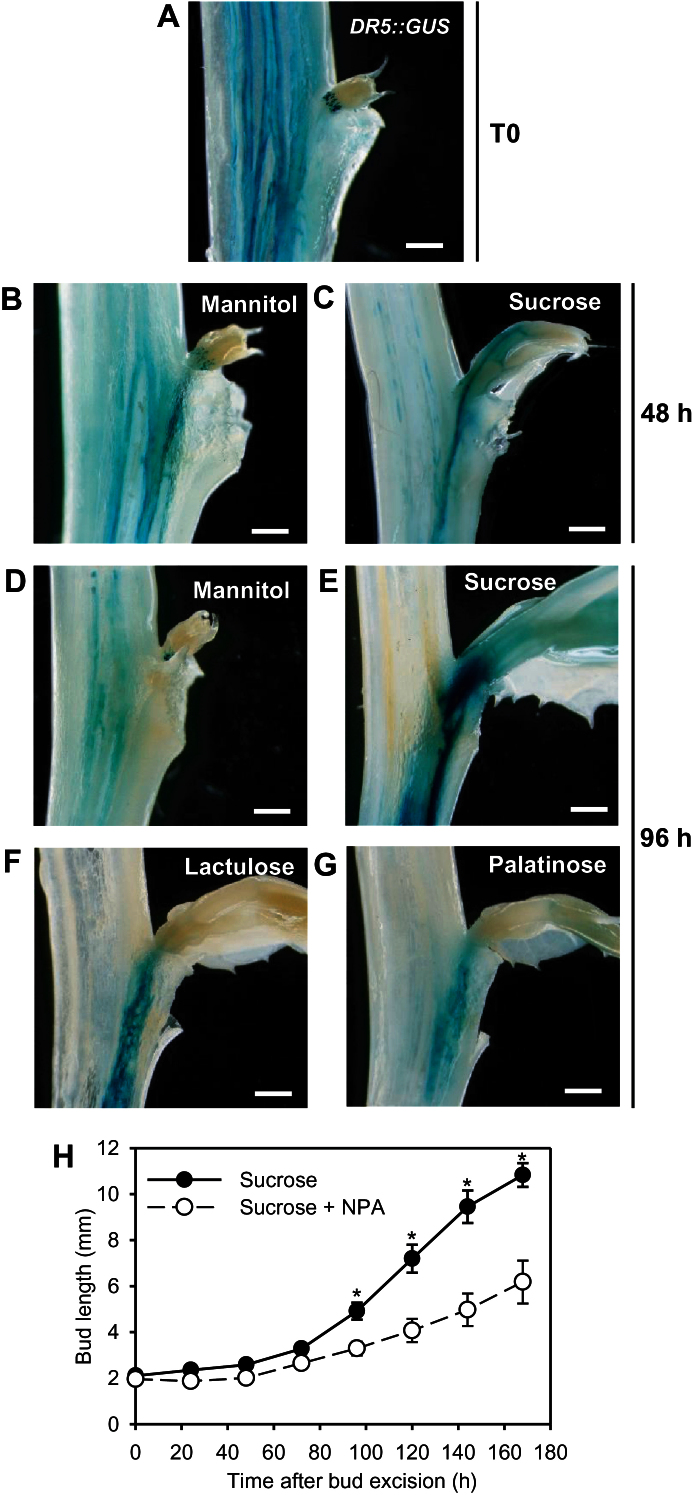
GUS staining in bud-bearing stem sections of the *DR5::GUS*-expressing pea line (A) before treatment (T0); or grown for 48h with 100mM (B) mannitol or (C) sucrose; or grown for 96h with 100mM (D) mannitol or (E) sucrose or 80mM (F) lactulose or (G) palatinose. (H) Elongation of buds grown with 30mM sucrose and treated with a drop of gelose on buds containing 1% PEG, 0.01% Tween-20, and 0.2% DMSO supplemented or not with 1mM NPA. (A–G) Images are representative of five replicates; (H) mean ± SE of eight replicates. Asterisks indicate significant differences between the treatment conditions. White bars represent 1mm.

It is well known that an active bud exports its own auxin in the stem (Li and Bangerth, 1999; Domagalska and Leyser, 2011). The positive impact of sucrose on the establishment of an auxin transport canal between bud and stem was checked using a *DR5::GUS*-expressing pea line containing an auxin-inducible promoter fused to the β-glucuronidase reporter gene (DeMason and Polowick, 2009). On intact plants (Fig. 3A, T0), the apex-derived auxin flux in the stem was visible but no clear flux was detected between bud and stem. 48h after bud-bearing stem excision from the plant, an auxin transport canal was visible between bud and stem with both mannitol and sucrose, but the blue precipitate was more intense with sucrose (Fig. 3B, C). At 96h, this canal had consistently disappeared with mannitol but had intensified with sucrose (Fig. 3D, E). Similarly to sucrose, lactulose and palatinose showed a sustained auxin canal between bud and stem (Fig. 3F, G), suggesting a possible signalling role for sucrose in this process.

The results above demonstrate that the ability of sucrose to sustain bud outgrowth is related to its ability to sustain auxin export. To test whether auxin export is limiting in the effect of sucrose on bud outgrowth, we used NPA, an inhibitor of auxin transport, specifically applied on the bud. Bud elongation was slowed down in the presence of sucrose + NPA compared to sucrose alone (Fig. 3H). At 7 days, bud length was about 43% less with NPA than without NPA, indicating that auxin export from the bud is limiting for sustained growth in the presence of sucrose.

### Impact of sucrose on auxin concentration and synthesis in buds

**Fig. 4.**
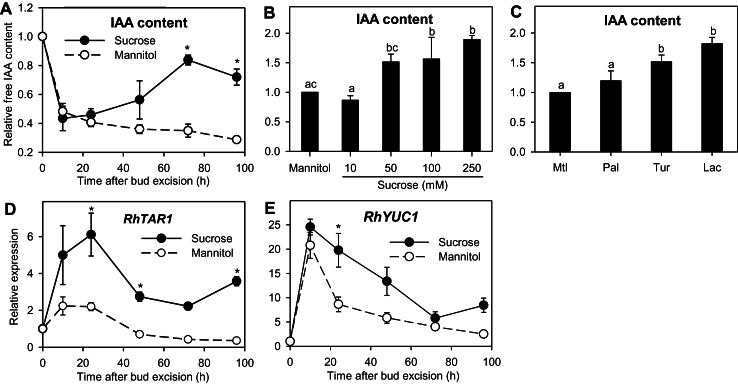
(A) Relative IAA content (relative to T0 value) in buds of *R. hybrida* grown *in vitro* with 100mM sucrose or 100mM mannitol for 96h. (B, C) Relative IAA content (relative to mannitol value) in buds grown for 48h with (B) 100mM mannitol or a range of sucrose concentrations (10, 50, 100, or 250mM), or (C) 100mM mannitol (Mtl) or 80mM sucrose analogues: palatinose (Pal), turanose (Tur), or lactulose (Lac). (D, E) Relative expression of (D) *RhTAR1* and (E) *RhYUC1*. Data are mean ± SE of three measurements on a pool of 60 buds. Asterisks and letters indicate significant differences between the different treatments for each time-point.

Sustained auxin export from buds implies an ability of the buds to synthesize their own auxin and transport it into the stem. In the first 10h, auxin levels dropped by a large amount in buds incubated with sucrose or mannitol (–57 and –52%, respectively; Fig. 4A). This level remained low for the buds kept on mannitol throughout the whole incubation period, while it gradually accumulated in the buds grown with sucrose, reaching its maximum level at 72h (+95% compared to 10h). The effect of sucrose on auxin accumulation was concentration dependent (Fig. 4B). Increasing sucrose to 50 or 100mM increased bud auxin content at 48h by about 56% compared to mannitol. The increase reached 89% with 250mM sucrose. Turanose and lactulose also increased the auxin content at 48h compared to mannitol (+51 and +82%, respectively; Fig. 4C), suggesting a possible signalling role of sucrose in this process.

Auxin is mainly synthesized from tryptophan through the indole-3-pyruvic acid (IPA) pathway (Mashiguchi *et al.*, 2011; Zhao, 2012) in two enzymatic steps implying the involvement of TRYPTOPHAN AMINOTRANSFERASE OF ARABIDOPSIS1 and related proteins (TAA1 and TARs) and YUCCA flavin monooxygenase-like proteins (Zhao, 2012; Ljung, 2013). To test whether auxin accumulation in sucrose-fed buds is related to a stimulation of auxin synthesis within the bud, we investigated the effect of sucrose on certain genes implied to be involved in auxin biosynthesis on the basis of their sequence: *RhTAR1* and *RhYUC1* (Supplementary Table S2). As for auxin content, the expression level of these genes was mainly stimulated in the buds supplied with sucrose compared to mannitol (Fig. 4D, E). Higher expression with sucrose compared with mannitol occurred as early as 10h for *RhTAR1* (25- vs 21-fold) and 24h for *RhYUC1* (6- vs 2-fold), and was maintained over the 96h period studied. The general temporal pattern of expression was close for the two genes, with an induction peak at 10h, followed by a decrease until 72h.

### Impact of sucrose on PIN abundance and polarization in buds

**Fig. 5.**
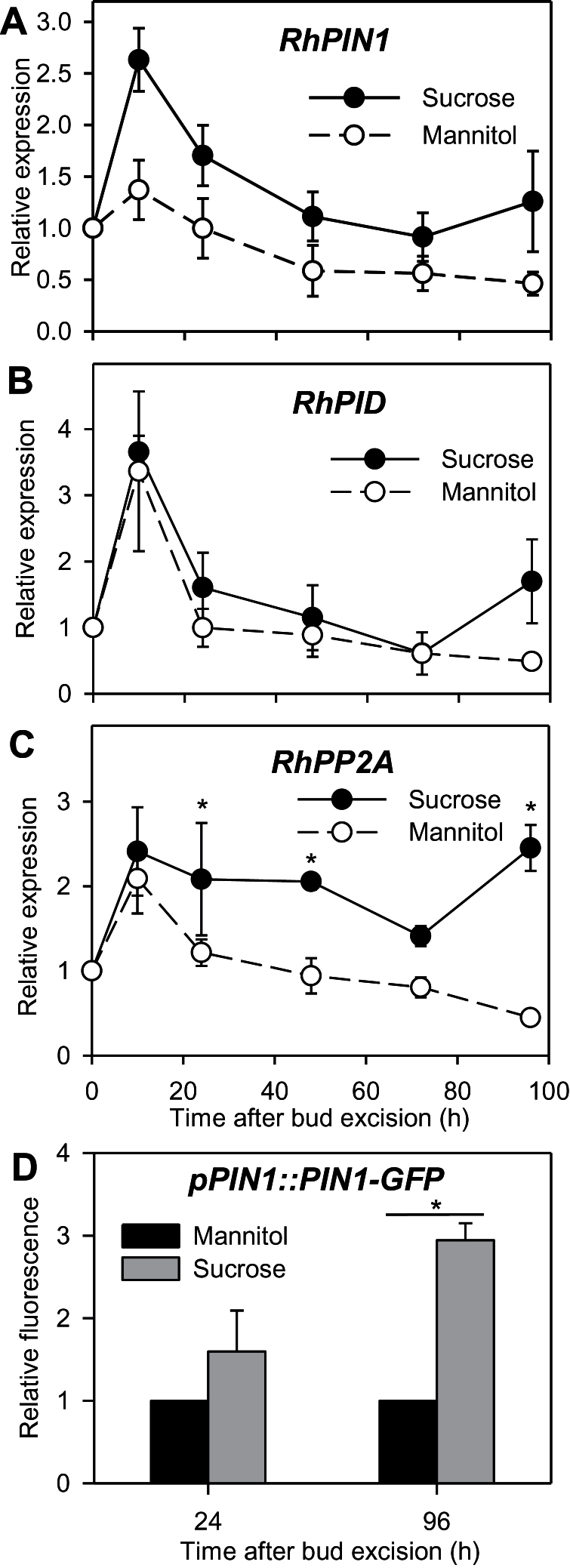
Relative expression of (A) *RhPIN1*, (B) *RhPID*, and (C) *RhPP2A* in buds grown *in vitro* for 96h with 100mM mannitol or sucrose. (D) Relative fluorescence of the GFP signal at the polarized pole of the cells in bud stems of the *pPIN1::PIN1-GFP*-expressing tomato line grown for 24h or 96h with 100mM mannitol or sucrose. Data are mean ± SE of three measurements on a pool of 60 buds (A–C) and four replicates (D). Asterisks indicate significant differences between the different treatments for each time point.

Intercellular auxin export is mainly ensured by PIN-FORMED efflux carrier proteins (Petrášek and Friml, 2009). Their targeting to a pole of the cell is antagonistically mediated by the PINOID serin/threonin protein kinase (PID) and the PROTEIN PHOSPHATASE 2A (PP2A) (Michniewicz *et al.*, 2007; Zhang *et al.*, 2010). The transcripts of *RhPIN1*, putatively encoding an AtPIN1-like auxin efflux carrier (Supplementary Table S2), rapidly accumulated in the buds within the first 10h (a 3-fold increase; Fig. 5), and were 2-fold higher with sucrose than with mannitol after 10h. The transcript levels of *RhPIN1* then decreased. *RhPID* and *RhPP2A* expression also peaked after 10h (4- and 2-fold increases, respectively) with mannitol and sucrose. However, upregulation with sucrose compared to mannitol only occurred late in the outgrowth process, after 96h for *RhPID* and after 24h for *RhPP2A*.

To check whether the presence of sucrose resulted in the targeting of PINs to the plasma membrane in the bud, we assessed the amount of PIN1 polarized in buds grown with sucrose or mannitol in the *pAtPIN1::AtPIN1-GFP* tomato line (Money Maker background). As in *R. hydrida*, sustained bud outgrowth in this tomato line was promoted by sucrose but not by mannitol (Supplementary Figure S3). A GFP signal localized at the basal pole of the cells was detected with both sugar treatments (Supplementary Figure S4). The quantification of GFP signal indicated that there was no significant difference between sucrose and mannitol at 24h, but that it was 3-fold higher with sucrose than with mannitol at 96h (Fig. 5D).

### Impact of sucrose on cytokinin synthesis and accumulation in stem tissues

**Fig. 6.**
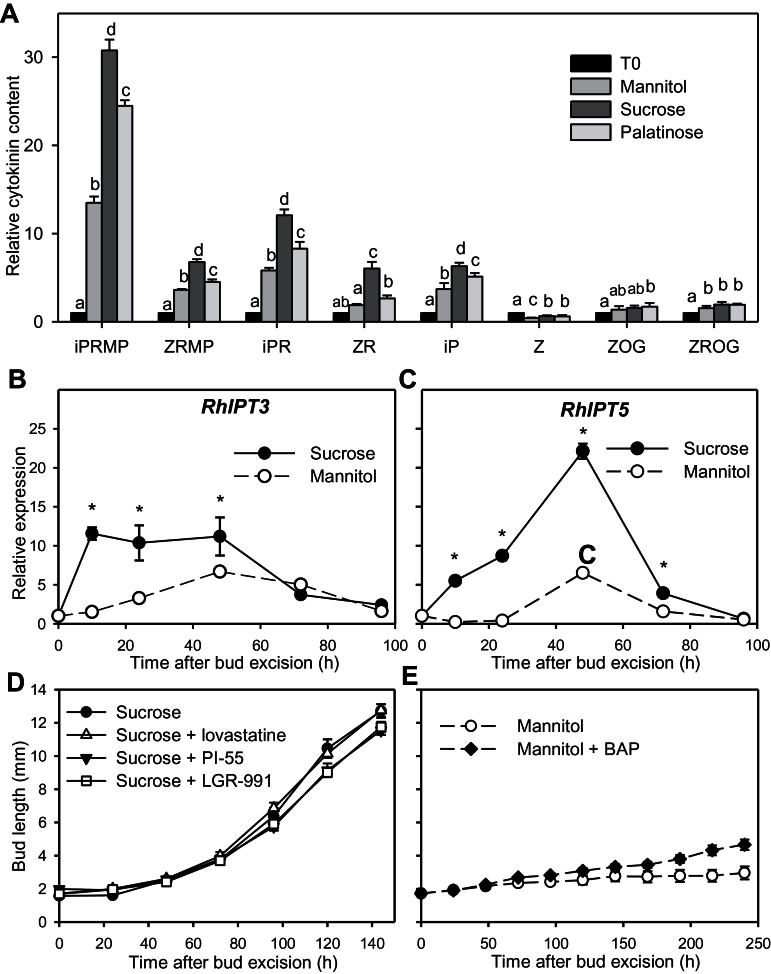
(**A**) Nodal stem content in isopentenyl adenine riboside 5′-monophosphate (iPRMP), zeatin riboside 5′-monophosphate (ZRMP), isopentenyl adenine riboside (iPR), zeatin riboside (ZR), isopentenyl adenine (iP), zeatin (Z), Z *O*-glucoside (ZOG), and ZR *O*-glucoside (ZROG) for intact plants at the FBV stage (T0) or grown for 24h on 100mM mannitol, 100mM sucrose, or 30mM palatinose; contents are expressed relative to T0. Relative expression is also shown of (B) *RhIPT3* and (C) *RhIPT5* in nodal stem sections grown *in vitro* with 100mM mannitol or 100mM sucrose up to 96h after their excision. Also elongation of buds grown with (D) 30mM sucrose alone or with 10 µM lovastatine, PI-55, or LGR-991; and (E) with 30mM mannitol alone or with 10 µM 6-benzylaminopurine (BAP). Data are mean ± SE of three measurements on a pool of 60 buds (A–C) and 10 replicates (D, E). Asterisks and letters indicate significant differences between the different treatments for each time point.

To test the involvement of cytokinins in sucrose-stimulated bud outgrowth, we quantified their accumulation in stem sections cultivated *in vitro* between 0 and 24h (which is before rapid bud growth) with mannitol, sucrose, or palatinose (Fig. 6A). Active cytokinins are derived from intermediate forms and can be converted into inactive conjugated forms (Sakakibara, 2006). The three types of sugars induced an accumulation of cytokinin intermediate forms (iPRMP, ZRMP, iPR, and ZR) and an active form (iP), while conjugated inactive forms (ZOG and ZROG) accumulated more modestly and zeatin, another active form, did not accumulate. Interestingly, cytokinin accumulation with mannitol was lower than with sucrose, and slightly lower than with palatinose. This demonstrates that cytokinins accumulate before rapid bud growth in the presence of sucrose.

The rate-limiting step of cytokinin biosynthesis is catalysed by enzymes of the ISOPENTENYL TRANSFERASE (IPT) family (Sakakibara, 2006; Miyawaki *et al.*, 2006). As a complement to cytokinin quantification at 24h, we quantified the relative expression of *R. hybrida Isopentenyl Transferase3* and *5* (*RhIPT3* and *RhIPT5*) in the stem segments (Fig. 6B and C). These genes code for proteins that share high homology with the cytokinin-biosynthesis enzymes of *Arabidopsis AtIPT3* and *AtIPT5*, respectively (Supplementary Table S2). During the first 48h, sucrose promoted the expression of *RhIPT3* and *RhIPT5* compared to mannitol. In response to sucrose, expression of *RhIPT3* rapidly increased just after stem excision, within the first 10h (12-fold increase), whereas in response to mannitol, it increased progressively and to a lesser extent than sucrose (7-fold increase). *RhIPT5* transcripts accumulated more progressively than *RhIPT3* with sucrose to reach their maximum value at 48h (22-fold increase), but strongly compared with mannitol (7-fold). After 48h, the expression level of *RhIPT3* and *RhIPT5* dropped to the same level with sucrose and mannitol. These results suggest that sucrose promoted cytokinin accumulation in the stems at least through a rapid upregulation of *RhIPT3* and *RhIPT5*.

To determine whether the impact of sucrose on bud outgrowth could be mediated by cytokinins, 10 µM solutions of different inhibitors of cytokinin synthesis (lovastatine) and perception (PI-55, LGR-991) were added to the growth medium in the presence of 30mM sucrose (Fig. 6D). On the whole, bud elongation was insensitive to these inhibitors. No difference was observed in the elongation of buds cultivated with sucrose or sucrose plus cytokinin inhibitors during the first 72h. At 96h, bud length became lower in the presence of PI-55 and LGR-991, but differences between treatments were slight and occurred well after buds entered rapid growth. Moreover, supplying 10 µM BAP, a synthetic cytokinin, to medium with mannitol did not trigger sustained bud outgrowth (Fig. 6E). These results suggest that the observed promoted effect of sucrose on rapid bud growth is not mediated by cytokinins.

### Impact of sucrose on strigolactone signalling genes

**Fig. 7.**
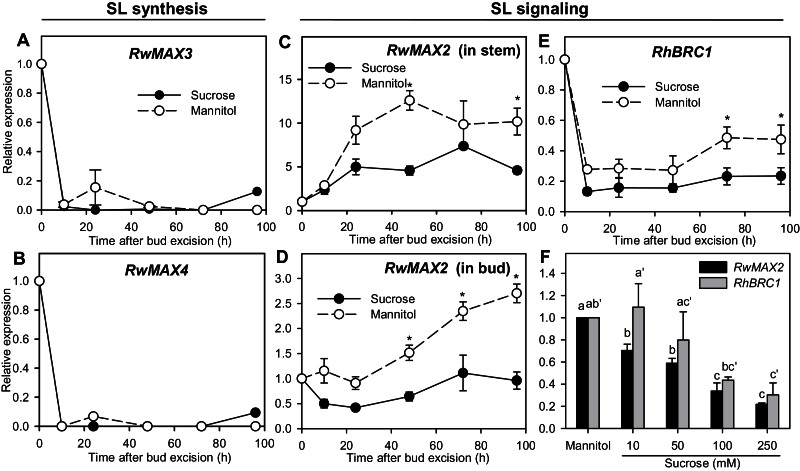
Relative expression of (A) *RwMAX3*, (B) *RwMAX4*, and (C) *RwMAX2* in nodal stems grown for 96h with 100mM mannitol or sucrose; relative expression of (D) *RwMAX2*, and (E) *RhBRC1* in buds grown for 96h with 100mM mannitol or sucrose; and (F) relative expression of *RwMAX2* and *RhBRC1* in buds grown for 24h with 100mM mannitol or 10, 50, 100, or 250mM sucrose. Data are mean ± SE of three replicates. Asterisks indicate significant differences between sucrose and mannitol for each time point. Letters indicate significant differences between means for each gene.

To test the involvement of strigolactones in sucrose-stimulated bud outgrowth, we quantified the expression of genes putatively implied in strigolactone synthesis and signalling. Strigolactones are produced by a sequential cleavage of a carotenoid precursor that involves different CAROTENOID CLEAVAGE DIOXYGENASE enzymes (CCD7/*MAX3/RMS5*, CCD8/*MAX4/RMS1*) (Lin *et al.*, 2009; de Saint Germain et al., 2013). Strigolactone perception is notably mediated by an F-box protein encoded by *RMS4/MAX2/D3* (Stirnberg *et al.*, 2002, 2007; Hamiaux *et al.*, 2012). The expression of *RwMAX3* and *RwMAX4*, putatively involved in strigolactone synthesis, dropped abruptly after excision to become undetectable after 10h and then remained very low whether the buds were cultivated with sucrose or mannitol (Fig. 7A, B). The expression of *RwMAX2* was repressed with sucrose compared to mannitol in both stems and buds (Fig. 7C, D). In stems, *RwMAX2* expression increased within the first 24h with both sucrose and mannitol, but to a lesser extent with sucrose (5-fold increase) than with mannitol (9-fold increase). Afterwards, *RwMAX2* expression in stems remained stable for both treatments. In buds, *RwMAX2* expression dropped (0.5-fold) within the first 24h for sucrose while it remained stable for mannitol. This initial downregulation of *RwMAX2* was negatively related to sucrose concentration and its expression was 5-fold lower with 250mM sucrose than with mannitol (Fig. 7F). After 24h, *RwMAX2* expression in buds increased to reach, at 96h, a value close to that observed at 0h with sucrose and a value 3-fold higher with mannitol.

Strigolactones modulate *BRC1* expression, a gene coding for a transcription factor that inhibits bud outgrowth and with expression well related to bud outgrowth state (Aguilar-Martínez *et al.*, 2007; Braun *et al.*, 2012). Similarly to *RwMAX2*, *RhBRC1* expression in buds was repressed overall with sucrose compared to mannitol (Fig. 7E). During the first 10h, irrespective of the sugar tested, *RhBRC1* expression showed a strong drop, which was slightly stronger with sucrose than with mannitol (8- and 4-fold, respectively). Thereafter, *RhBRC1* expression remained quite stable until 48h and then, with mannitol only, slightly re-increased. *RhBRC1* expression at 24h was negatively related to sucrose concentration, exhibiting much lower values with 100 or 250mM sucrose compared to mannitol, 10, or 50mM sucrose (Fig. 7F).

## Discussion

### Sucrose promotes sustained bud outgrowth

Bud outgrowth can be divided into three stages: a dormancy stage, a transition stage, and a sustained growth stage (Stafstrom and Sussex, 1992; Devitt and Stafstrom, 1995; Cline, 1997; Dun *et al.*, 2006). Depending on the conditions, buds in the transition stage will either go back to dormancy or enter sustained growth (Waldie *et al.*, 2010). We clearly observed two growth periods for *R. hybrida* buds after their excision from the bud-bearing stem segments and their transfer *in vitro*: an initial period of slow growth, which indicates that buds are not in a dormant stage, followed by a phase of rapid elongation in response to sucrose supply (Fig. 1). There was a progressive delay in buds entering rapid elongation when sucrose availability decreased. With mannitol, used as the ‘no sugar’ control, buds stopped their growth after the initial slow growth phase. These results support the hypothesis that sugar availability controls the entrance of buds into sustained growth. This need for sugar is conserved across species, as demonstrated in *Arabidopsis* and *P. sativum* (Fig. 2). Our results complement those showing that early bud release after decapitation is also controlled by an increase in sugar availability in pea (Mason *et al.*, 2014). For *in vitro*-cultivated *R. hybrida* buds, we observed a rapid degradation of starch reserves in the stem parenchyma (Supplementary Figure S5A) after excision of bud-bearing stem segments from the plant. Starch degradation may increase sugar availability for buds and in turn lead to their initial slow growth. Alternatively, initial slow bud growth could be related to the high auxin drop in the stem observed as early as 10h after stem excision (Supplementary Figure S6).

### Sucrose acts as a trophic and signalling entity in bud outgrowth

The role of sucrose cannot be restricted to a trophic role since different non-metabolizable sucrose analogues induced bud elongation in *R. hybrida, Arabidopsis*, and *P. sativum in vitro* (Fig. 2). Bud elongation was not always similar between sucrose and the analogue tested, probably due to different conformations of analogues, as previously observed with barley embryos (Loreti *et al.*, 2000) and in tobacco cell suspensions (Atanassova *et al.*, 2003). By contrast to sucrose, none of the sucrose analogues increased the dry weight of the bud-bearing stem segments, demonstrating that none of them were metabolized (Supplementary Figure S5B), which is in line with previous studies (Loreti *et al.*, 2000; Atanassova *et al.*, 2003). Moreover, the analogues were not absorbed by *RhSUC2* (Supplementary Table S4; Rabot *et al.*, 2012), a sucrose transporter preferentially expressed in *R. hybrida* during bud outgrowth (Henry *et al.*, 2011). Such an absence of analogue import into cells rules out their potential degradation by vacuolar invertases and sucrose synthases, as reported with palatinose in sugarcane cells (Wu and Birch, 2011). Finally, the effect of analogues was unlikely to be an indirect result of an impact on starch reserve mobilization or auxin depletion from the stem, as starch and auxin decreases in the stem were similar among treatments with and without analogues (Supplementary Figure S5A and C). These findings and others, discussed below, are consistent with the fact that sugars can serve, at least partly, as signalling molecules in the control of bud outgrowth.

Sucrose promotes sustained auxin synthesis within buds and auxin export from bud to stem. It is well known that bud outgrowth occurs along with auxin export out of the bud (Morris, 1977; Li and Bangerth, 1999). We checked this for sucrose- or sucrose analogue-promoted bud outgrowth using the auxin-inducible *DR5::GUS*-expressing pea line (Fig. 3A–G). We observed a sustained auxin canal between bud and stem with sucrose or sucrose analogues, but not with mannitol. Interestingly, we noted initial auxin export just after excising the bud-bearing stem segments, irrespective of the presence of sucrose or mannitol. Consistently, we noted a substantial auxin drop in buds within the first 10h after stem excision with sucrose or mannitol (Fig. 4A). Such a drop could be explained as follows: stem excision from the plant is followed by a rapid auxin drop in the stem (Supplementary Figure S6), initiating the creation of a canal of active auxin transport between bud and stem by positive feedback between auxin directional flux and PIN polarization in the direction of the flux (Bennett *et al.*, 2014). In line with this idea, the genes responsible for PIN polarization, *RhPID* and *RhPP2A*, were rapidly induced after stem excision with sucrose or mannitol (Fig. 5B, C).

The maintenance of auxin export with sucrose or its non-metabolizable analogues was accompanied by the capacity of the bud to synthesize auxin. After the initial auxin drop due to excision, buds supplied with either sucrose or non-metabolizable analogues were able to re-accumulate auxin, in contrast to buds grown with mannitol (Fig. 4A). Stimulation of auxin synthesis by sugars has been reported in a variety of developing plant organs including kernel embryos (LeClere *et al.*, 2010), as well as *Arabidopsis* roots (Mishra *et al.*, 2009), seedlings (Sairanen *et al.*, 2012), and hypocotyls (Stewart Lilley *et al.*, 2012). *RhTAR1* and *RhYUC1*, two genes involved in the indole-3-pyruvic acid pathway, the main auxin biosynthesis pathway (Mashiguchi *et al.*, 2011), showed a higher expression with sucrose compared with mannitol; this began early, before any visible effect on auxin re-accumulation in buds (Fig. 4D, E). This induction of auxin synthesis genes by sucrose could be direct via the WRKY transcription factor (TF) from *R. hybrida*, SUCROSE SIGNALLING1 (RhSUSI1). Indeed, this orthologue of the sucrose-induced TF SUSIBA2 in barley (Sun *et al.*, 2003) was both induced by sucrose in the bud and able to bind to the promoter of *RhTAR1* (Supplementary Figure S7), suggesting that sucrose could induce *RhTAR1* in an RhSUSI1-dependent manner. For buds cultivated with mannitol, we observed an initial peak in auxin biosynthesis gene expression, as with sucrose, but expression values progressively decreased to become even lower than those observed with intact plants. This initial peak may be related to the release of sucrose and hexoses from the breakdown of stem starch reserves, as well as to auxin depletion in the stem after excision (Supplementary Figures S5 and S6).

Besides auxin synthesis, the maintenance of an auxin canal with sucrose was also highly related to the capacity of buds to upregulate PIN1, an auxin efflux carrier involved in polar auxin transport and auxin canalization (Wisniewska *et al.*, 2006; Bennett *et al.*, 2014). *RhPIN1* showed an early higher expression with sucrose compared with mannitol, which was maintained throughout the 96-h period studied (Fig. 5A). Consistent with this, glucose highly upregulates *AtPIN1* and *AtPIN2* in *Arabidopsis* roots (Mishra *et al.*, 2009) and sucrose upregulates *AtPIN7* and downregulates *AtIAA3*, a negative regulator of AtPIN7 in *Arabidopsis* shoots (Stewart Lilley *et al.*, 2012). *RhPP2A*, which is required for the posttranslational mechanisms leading to the polarization of PIN proteins at the basal pole of the cells (Michniewicz *et al.*, 2007; Grunewald and Friml, 2010), was also upregulated with sucrose. However, we did not observe sucrose-mediated *RhPP2A* upregulation as early as *RhPIN1* upregulation (Fig. 5C), suggesting that *RhPP2A* may be involved in the maintenance of the canal rather than in its initial establishment. Consistently, in *pPIN1::PIN1-GFP*-expressing tomato, sucrose triggered a higher GFP signal than mannitol at the basal pole of cells in bud stem tissues during the sustained bud outgrowth period (after 96h; Fig. 5D). These findings suggest that sucrose could promote the establishment of polar auxin transport within the axillary bud by controlling PIN protein synthesis and/or PIN targeting to the plasma membrane. Such regulation could be the result of the negative impact of sucrose on the strigolactone pathway (discussed below), which has been shown to deplete PIN proteins from the plasma membrane (Shinohara *et al.*, 2013). Moreover, it cannot be excluded that the effect of sucrose on PIN1 polarization is the consequence of an increase of the auxin flow between bud and stem due to stimulation of auxin synthesis.

Altogether, these findings support the hypothesis that the sustained bud outgrowth observed in response to sucrose involves an early stimulation and the maintenance of processes responsible for auxin synthesis and transport. By applying NPA, an inhibitor of auxin transport, specifically on buds we further demonstrated that auxin export from the bud is limiting in sucrose-promoted sustained bud outgrowth (Fig. 3H). Similarly, NPA application on pea buds of decapitated plants was able to inhibit sustained bud outgrowth, but not earlier phases (Brewer *et al.*, 2009; Mason *et al.*, 2014), suggesting that auxin transport in bud is not critical in bud release following decapitation, although it may be crucial for ongoing bud outgrowth (Dun *et al.*, 2006; Ferguson and Beveridge, 2009).

### Sucrose promotes sustained growth in a cytokinin-independent manner

Cytokinins are involved in stimulation of bud outgrowth in different species including rosebush (Sachs and Thimann, 1967; Bredmose *et al.*, 2005; Shimizu-Sato *et al.*, 2009). Our results demonstrate that sucrose upregulates cytokinin biosynthesis in stem tissues and that a disaccharide signalling pathway can be at least partially involved. Indeed, compared to mannitol, sucrose and palatinose induced an overaccumulation of intermediate and active forms of cytokinins in *R. hybrida* stems very early in the outgrowth process, and the overexpression of *RhIPT3* and *RhIPT5*, two cytokinin biosynthesis-related genes (Fig. 6A–C). The effect of sugar on cytokinin production has previously only been reported for *Lilium* floral tissues (Arrom and Munné-Bosch, 2012a) and *Arabidopsis* seedlings (Kushwah and Laxmi, 2013). The effect of sucrose on the expression of *RhIPT3* and *RhIPT5* started after stem excision and was maintained over the first 48h during the phase of slow growth (Fig. 1B). Thereafter, their expression levels dropped (Fig. 6B, C), as previously observed with *PsIPT1* and *PsIPT2* in *P. sativum* stems upon decapitation (Tanaka *et al.*, 2006). This may involve a feedback loop in which auxin derived from outgrowing buds after 48h represses cytokinin biosynthesis, as suggested by the expression pattern of the cytokinin-catabolizing *R. hybrida CYTOKININ OXIDASE/DEHYDROGENASE1*, *RhCKX1* (Supplementary Figure S8), an auxin-inducible gene in stems (Shimizu-Sato *et al.*, 2009). *RhCKX1* expression was low in the first 48h following excision and subsequently increased only in the presence of sucrose.

Beside sugars, cytokinin biosynthesis is well known to be repressed by auxin in different species (Tanaka *et al.*, 2006; Minakuchi *et al.*, 2010), while it is induced by nitrogen in *Arabidopsis* (Takei *et al.*, 2004). This makes cytokinin production in the nodal stem a good potential integrator of the nutrient and auxin status in the regulation of bud outgrowth. However, we report here that cytokinins are not necessarily involved in sucrose-promoted bud outgrowth in our system. Indeed, bud outgrowth with sucrose was not inhibited by different cytokinin synthesis and perception inhibitors (Fig. 6D). Moreover, the addition of BAP, a synthetic cytokinin, in the medium did not trigger rapid growth of buds grown with mannitol, indicating that cytokinins are not limiting in our system (Fig. 6E). Similarly, cytokinins applied directly to axillary buds or the overexpression of cytokinin biosynthesis genes does not always induce bud outgrowth in other species (King and Van Staden, 1988; Medford *et al.*, 1989).

### Sucrose downregulates *MAX2* and *BRC1*, two genes involved in strigolactone signal transduction

Strigolactones are involved in the inhibition of bud outgrowth and have been suggested to be second messengers of auxin (Brewer *et al.*, 2009; Waldie *et al.*, 2010, 2014). In buds, the expression of the strigolactone synthesis-related genes, *RwMAX3* and *RwMAX4*, was not modulated by sucrose (Fig. 7A, B), but it dropped a lot within the first 10h, probably related to auxin depletion following stem excision. In contrast, *RwMAX2*, a key regulatory gene in the signal transduction of strigolactones, was repressed early and in a concentration-dependent manner by sucrose (Fig. 7C, D, F). Strigolactone perception through *RwMAX2* could thus be one of the ways whereby sucrose promotes sustained bud outgrowth. *MAX2* was also reported to be involved in bud outgrowth regulation by light. In sorghum, inhibition of bud outgrowth in the *phyB* mutant or by FR treatment was related to high *SbMAX2* expression in buds (Kebrom *et al.*, 2010). In *Rosa*, darkening of the distal part of the shoot triggered a strong increase of *RwMAX2* expression in darkened buds (Djennane *et al.*, 2014).

*BRC1* appears as a common target for different signals controlling bud outgrowth, including strigolactones and cytokinins, and thus as an essential component of bud outgrowth control (Aguilar-Martínez *et al.*, 2007; Braun *et al.*, 2012; Dun *et al.*, 2012). The expression of *R. hybrida* BRANCHED1, *RhBRC1*, in buds was repressed early with sucrose compared to mannitol, and with high sucrose concentrations (100, 250mM) compared with low concentrations (10, 50mM; Fig. 7E, F). Accordingly, Mason *et al.* (2014) observed that artificially increasing sucrose levels in pea plants repressed *BRC1* expression early. In our conditions, *RhBRC1* expression appeared more sensitive to stem excision than to external sucrose supply. Indeed, the expression showed an initial strong drop whether the buds were cultivated with sucrose or mannitol. This drop may be related to the fact that stem excision increased sucrose and hexoses from breakdown of stem starch reserves, or to the auxin depletion from the stem, another variable known indirectly to control *BRC1* (Chen *et al.*, 2013) (Supplementary Figures S7 and S8).

In conclusion, our study demonstrates that sucrose, recently shown to control initial bud release, also regulates the entrance of buds into sustained growth. We identified several hormonal components of the bud outgrowth regulatory network that were affected early by sucrose availability before the effect of sucrose was visible on bud outgrowth, suggesting their possible involvement in the control of sustained bud growth (summarized in Supplementary Figure S9). This study on isolated buds provides basic information on which further investigations could focus for understanding the mechanisms whereby sugars control bud outgrowth and therefore their role in the control of bud outgrowth patterns *in planta*.

## Supplementary material

Supplementary data can be found at *JXB* online.

Supplementary Table S1. Parameters used for cytokinin content analysis.

Supplementary Table S2. Putative identity and functions of the genes cloned in this study.

Supplementary Table S3. Primers used for qRT-PCR.

Supplementary Table S4. Impact of sucrose analogues on RhSUC2 transport capacity in yeast.

Supplementary Figure S1. Elongation time course of buds grown with sucrose or sucrose plus mannitol, showing mannitol toxicity.

Supplementary Figure S2. Observed and fitted elongation time courses for buds.

Supplementary Figure S3. Impact of sucrose on tomato bud elongation.

Supplementary Figure S4. Micrographs of the *DR5::GFP*-expressing tomato bud stems.

Supplementary Figure S5. Metabolization of sucrose analogues in *R. hybrida*.

Supplementary Figure S6. Auxin depletion in stem segments.

Supplementary Figure S7. Relative expression of RhSUSI with sucrose or mannitol.

Supplementary Figure S8. Relative expression of *RhCKX1.*

Supplementary Figure S9. Schematic representation of the impact of sucrose on the hormonal regulation of bud outgrowth.

Supplementary material. Additional experimental details.

Supplementary Data
