# Supplementary material for: Sucrose is an early modulator of the key hormonal mechanisms controlling bud outgrowth in Rosa hybrida
Source: J Exp Bot. 2015 Apr 13;66(9):2569–82. doi: 10.1093/jxb/erv047 (PMC4986866; doi:10.1093/jxb/erv047)
Supplement: Supplementary Data [file supp_66_9_2569__index.html]

Sucrose is an early modulator of the key hormonal mechanisms controlling bud outgrowth in Rosa hybrida — Supplementary Data 

# Sucrose is an early modulator of the key hormonal mechanisms controlling bud outgrowth in *Rosa hybrida*

## Supplementary Data

Data files

**Files in this Data Supplement:**

- Supplementary Data - Supplementary Data
