# Supplementary material for: Sucrose is an early modulator of the key hormonal mechanisms controlling bud outgrowth in Rosa hybrida
Source: J Exp Bot. 2015 Apr 13;66(9):2569–82. doi: 10.1093/jxb/erv047 (PMC4986866; doi:10.1093/jxb/erv047)
Supplement: Supplementary Data [file supp_erv047_erv047_Supp_data_05_02_2015.pdf]

SUPPLEMENTARY DATA

TITLE:
Sucrose is an early modulator of the key hormonal mechanisms controlling bud outgrowth in *Rosa hybrida*

Running title: Sucrose regulates bud outgrowth hormonal network

Authors:
François Barbier, Thomas Péron, Marion Lecerf, Maria-Dolores Perez-Garcia, Quentin Barrière, Jakub Rolcík, Stéphanie Boutet-Mercey, Sylvie Citerne, Hanaé Roman, Nathalie Leduc, Jérémy Lothier, Rémi Lemoine, Benoît Porcheron, José Le Gourrierec, Jessica Bertheloot, Soulaiman Sakr

Supplementary Table S1

A

Hormone	Retention time (min)	Ions mode	MSMS transition	Collision Energy (V)	Sampling cone voltage (V)
Z7G<br>$^{2}H_{5}$-t-Z7G	2.41	-	382>220<br>387>225	35	20
ZOG<br>$^{2}H_{5}$-t-ZOG	2.54	-	382>220<br>387>225	35	20
Z<br>^{15}N-t-Z	2.67	-	221>137<br>220>136	45	15
Z9G<br>$^{2}H_{5}$-t-Z9G	2.88	-	382>220<br>387>225	35	20
ZROG<br>$^{2}H_{5}$-t-ZROG	3.43	-	514>220<br>519>225	40	23
ZR<br>$^{2}H_{5}$-t-ZR	3.71	-	352>220<br>357>225	40	17
ZRMP<br>$^{2}H_{5}$-t-ZRMP	3.82	-	432>220<br>437>225	40	20
iPRMP<br>$^{2}H_{5}$-iPRMP	4.25	-	416>204<br>422>210	40	20
iP<br>$^{2}H_{5}$-iP	4.76	-	204>136<br>210>137	35	12
iPR<br>^{15}N-iPR	5.88	-	336>204<br>334>205	40	15

B

Hormone	LOQ ng/g DW	LOD ng/g DW
Z7G	ND	ND
ZOG *	8.66	2.6
Z	1.43	0.4
Z9G	ND	ND
ZROG *	2.85	0.86
ZR	1.11	0.33
ZRMP	11.71	3.51
iPRMP	2.99	0.9
iP	0.42	0.12
iPR	0.83	0.25

Supplementary Table S1. (**A**) Parameters used for LC-ESI-MS/MS analysis of cytokinin contents. (**B**) Summary of LOD and LOQ in samples (* in CKs g^{-1} DW).

Supplementary Table S2

**Protein identified in *Rosa hybrida***	**Peptidic homology with *Arabidopsis thaliana***		**Conserved domains characteristic of the putative protein functions**
**RhIPT3** (KJ933389)	AtIPT3 (AT3G63110)	Identities : 59 %	- Adenylate isopentenyltransferase domains
**RhIPT5** (KJ933390)	AtIPT5 (AT5G19040)	Identities : 68 %	- Adenylate isopentenyltransferase domains
**RhCKX1** (RC001490*)	AtCKX1 (AT2G41510)	Identities : 65 %	- FAD and cytokinin binding domain<br>- cytokinin dehydrogenase domain
**RhTAR1** (KJ933382)	AtTAR2 (AT4G24670)	Identities : 61 %	- Aminotransferase domains
**RhYUC1** (KJ933383)	AtYUC4 (AT5G11320)	Identities : 70 %	- NAD(P)-binding Rossmann-like domain<br>- FAD binding domain<br>- Flavin-binding monooxygenase-like<br>- Flavin-containing monooxygenase FMO GS-OX
**RhPIN1** (KJ933386)	AtPIN1 (AT1G73590)	Identities : 73 %	- Auxin Efflux Carrier (AEC) Family<br>- Membrane transport protein
**RhPID** (KJ933387)	AtPID (AT2G34650)	Identities : 81 %	- Serine/threonine protein kinase
**RhBRC1** (KF530803)	AtBRC1 (AT3G18550)	Identities : 71 %	- Transcription factor TCP24 (TEOSINTE BRANCHED1, CYCLOIDEA, AND PCF FAMILY 24)
**RhSUSI1** (KJ933388)	AtWRKY20 (AT4G26640)	Identities : 55 %	- 2 WRKY DNA-binding domains<br>- 1 Nuclear Localization Signal (NLS)

Supplementary Table S2. Homologies between the protein sequences of the genes cloned in *Rosa hybrida* and those of *Arabidopsis thaliana*, and conserved domains detected on the protein sequences of *Rosa hybrida* that could be implied in their putative function. Multiple sequence alignment was first performed with the BLAST tool of The Arabidopsis Information Resource site (TAIR, arabidopsis.org) and conserved domains were determined with the CDD tool of the NCBI or with NLS mapper. Accession code is given for each gene. * cluster number from *Rosa chinensis* EST data base (https://iant.toulouse.inra.fr/plants/rosa/FATAL).

Supplementary Table S3

Gene	Primers
RhIPT3	Fwd : CCACCATTTGCTTGGAGTT<br>Rev : CTCCAACGATGA TTGGAAGC
RhIPT5	Fwd : GGCACAGTGGATCCTGAGTC<br>Rev : CGTACTTCCTCCGAAACTCG
RhCKX1	Fwd : CCAGTCAACAAAACCAAGTGGAA<br>Rev : CCTGTGGAAGATGGCACCAC
RhBRC1	Fwd : TGCATTGTTTAACCCTCTTGCA<br>Rev : GTTCTTTCTCTTGTCTCGCTCTT
RwMAX2	Fwd : GCTGCCTATCCCGTTTTCCTC<br>Rev : AATCCCACAGTATCACCACAATCC
RwMAX3	Fwd : CAGCAGCTTCTTCGGGTTCT<br>Rev : CTTCACCACCATGTCAAATGGA
RwMAX4	Fwd : CCCGTGTTGGCGTTATCG<br>Rev : AGTTATGCTACCGTGAGTTCTC
RhTAR1	Fwd : TGTTTAGTCTGCCTGACTTTGCA<br>Rev : GGCTGCGTCTCCTGTTGAA
RhYUC1	Fwd : CGGTTGGCTTCGGAAGAA<br>Rev : TTGCTATCATAACGGCGTCGTA
RhPIN1	Fwd : ACCAAAGATCATAGCATGTG<br>Rev : ACAAATGGGACAATTCCTTG
RhPID	Fwd : GGGCGTTCGATTACTTTTGA<br>Rev : CTGGCAGGACTGTCTGAACA
RhPP2A	Fwd : TGTCACTGCATCAAAGGACAG<br>Rev : GACGAATTGTCTTCTCCACCA
RhSUSI1	Fwd : GCCTTGATCTTGGTGTTGGAA<br>Rev : TCGCAGGTGATTGTTGGATCT

Supplementary Table S3. Primers used for quantitative real time PCR (Q-PCR).

Supplementary Table S4

Sugar	Absorption	Absorption reduction (%)
Control	28,86	
10 mM sucrose	16,38	43,24
30 mM sucrose	9,57	66,84
10 mM turanose	27,83	3,57
30 mM turanose	25,36	12,13
10 mM lactulose	29,55	-2,39
30 mM lactulose	26,32	8,80
10 mM melibiose	20,99	13,34
30 mM melibiose	26,84	7,00

Supplementary Table S4. Sucrose uptake (3 mM 14C-sucrose) was measured in yeast transformed with either pDR/RhSUC2 or pDR alone (background) in the presence of different sugars. Results were initially expressed as RhSUC2-dependent uptake (uptake by RhSUC2-expressing yeast minus uptake by pPDR-expressing yeast, in nmol min-1 mg-1 protein), and then converted into percentages of the control uptake in the absence of sugar (100% or 28,86 nmol min-1 mg-1 protein). Results are the means of 3 independent experiments with 4 replicates *per* experiment.

Supplementary Figure S1

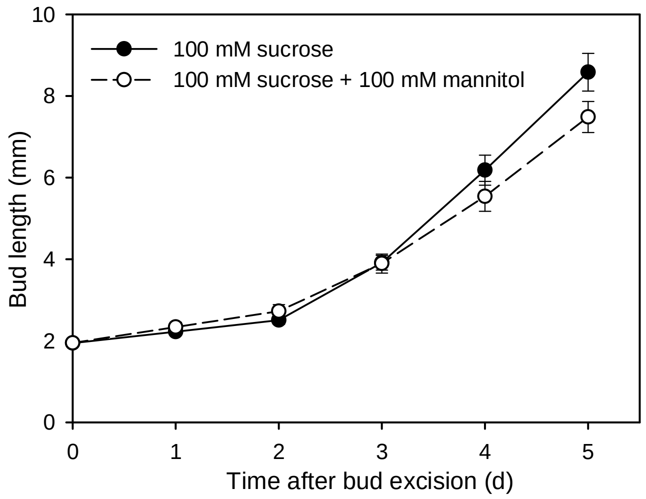

Supplementary Figure S1. Elongation time course of buds grown with 100 mM sucrose (black circles) or 100 mM sucrose combined with 100 mM mannitol (white circles). Data are the means ± s.e. of n=10 replicates.

Supplementary Figure S2

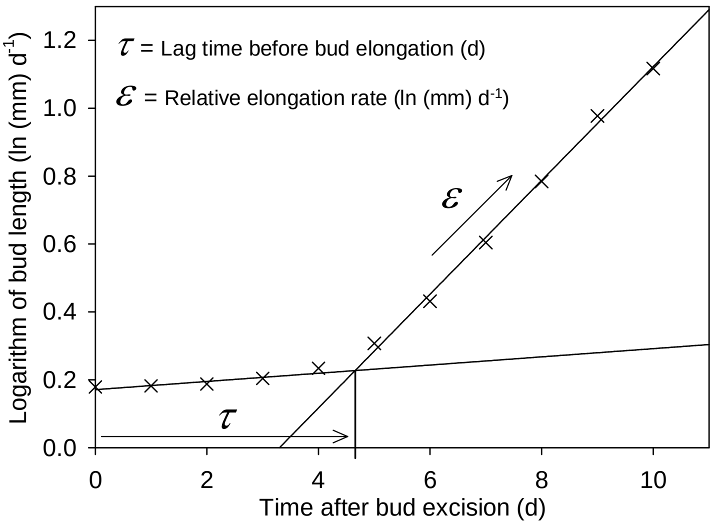

Supplementary Figure S2. Observed (crosses) and fitted (lines) elongation time courses for buds of nodal position 6 cultivated *in vitro* on classical MS medium after excision from *Rosa hybrida* primary axis at the FBV stage (time zero). Elongation is expressed in logarithmic scale. τ ï the lag time before rapid bud elongation ï and ε ï the relative elongation rate ï are used to characterize bud elongation.

Supplementary Figure S3

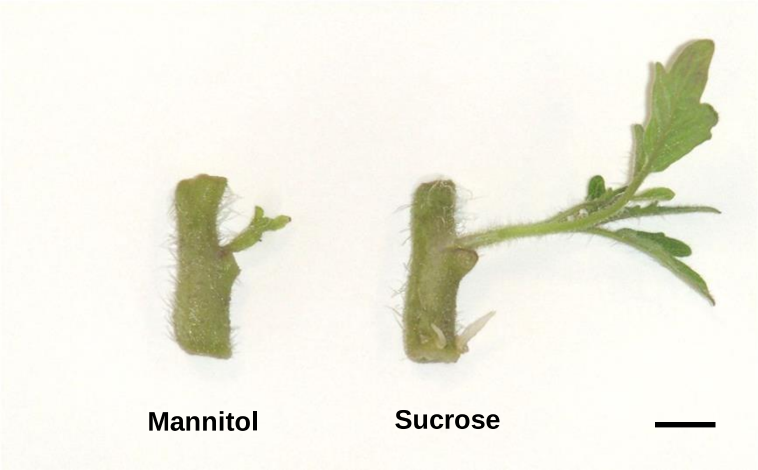

Supplementary Figure S3. Impact of 100 mM mannitol or sucrose after 8 days of treatment on bud elongation of *Solanum lycopersicum* ôMoney Makerô cultivated *in vitro*. The picture is representative of 5 to 6 replicates. Scale bar: 1 cm.

Supplementary Figure S4

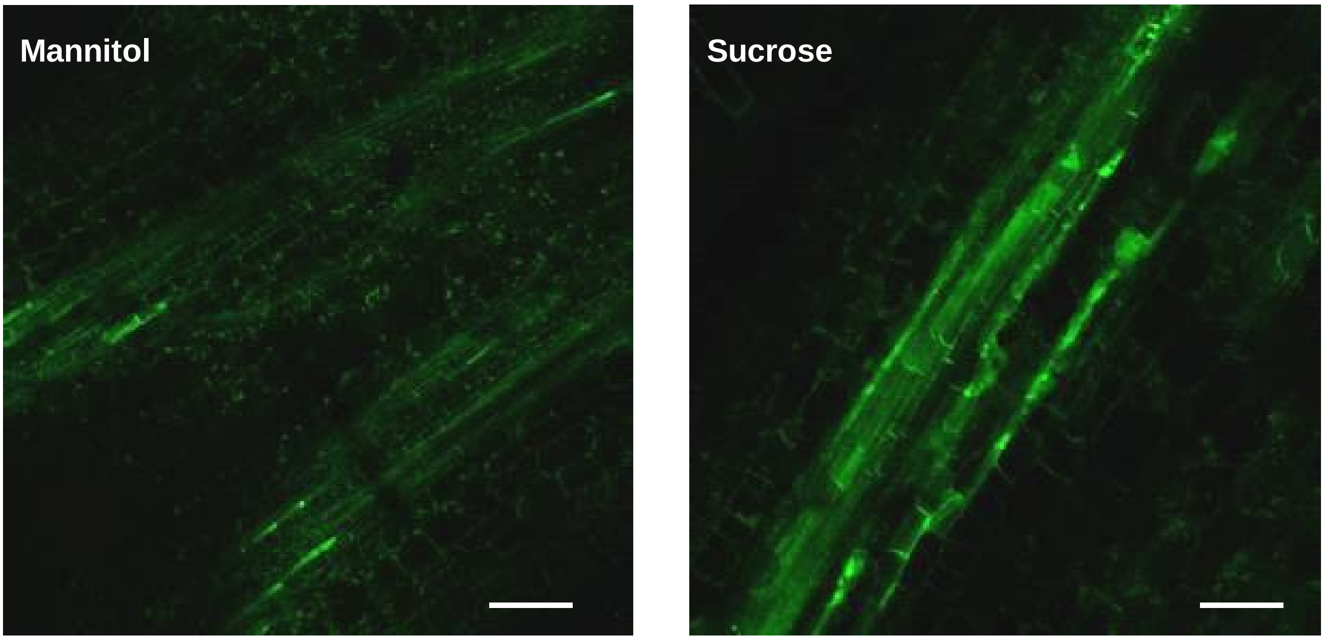

Supplementary Figure S4. PIN1 localization in bud stems of the *pPIN1::PIN1-GFP*-expressing tomato line grown 96 h with 100 mM mannitol (left) or sucrose (right). Micrographs are representative of 3 to 4 replicates. White bars represent 50 µm.

Supplementary Figure S5

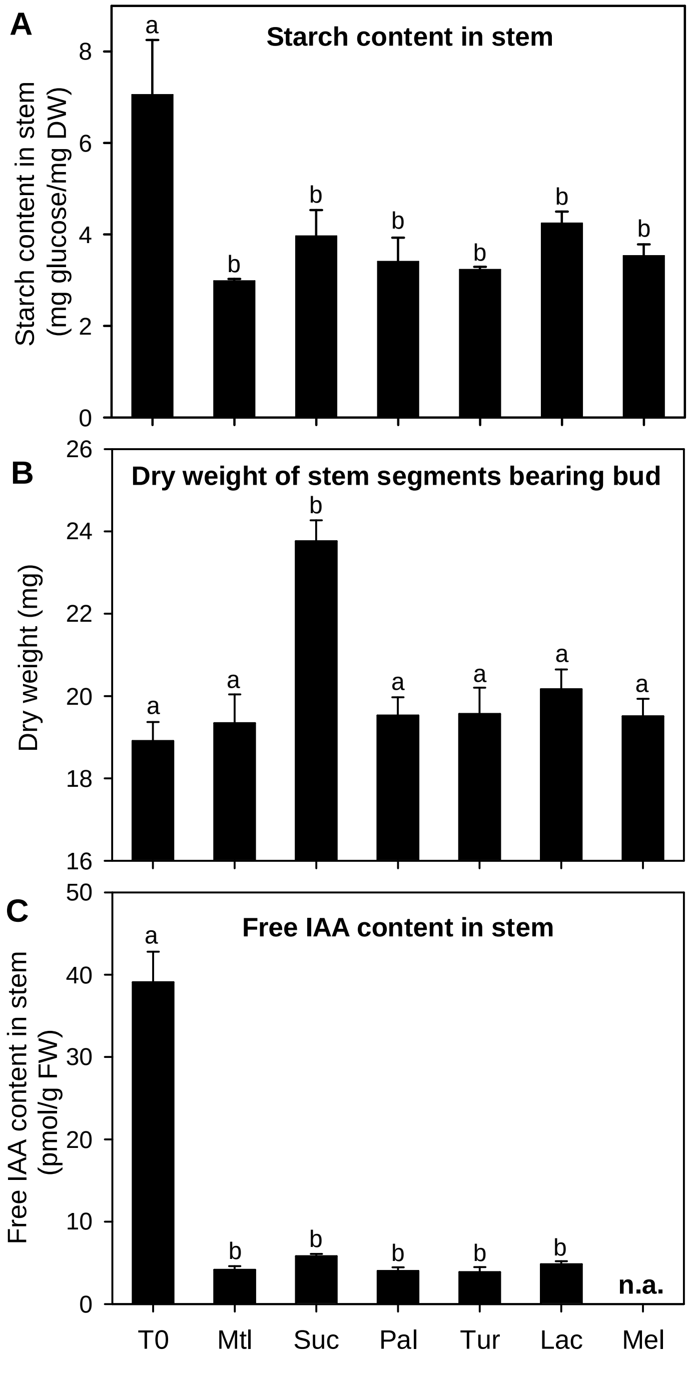

Supplementary Figure S5. (**A**) Stem starch content, (**B**) dry weight and (**C**) stem auxin content of bud-bearing stem segments grown for (**A**) 72 h or (**B**) and (**C**) 48 h with 80 mM mannitol (Mtl), sucrose (Suc), palatinose (Pal), turanose (Tur), lactulose (Lac) or melibiose (Mel). Data are the means ± s.e. of n=9 replicates for (**A**) and n=3 replicates for (**B**) and (**C**). Letters indicate significant differences (Ŭ= 0.05). The statistical analysis in (**B**) and (**C**) separate the values at 48 h from those at T0.

Supplementary Figure S6

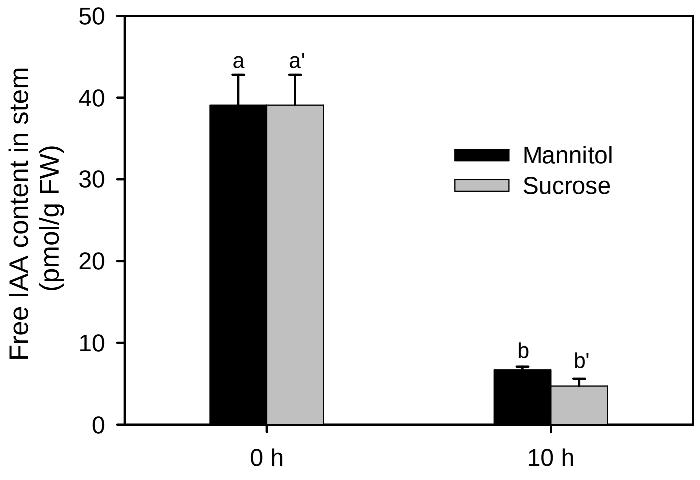

Supplementary Figure S6. Free IAA content in stem segments of intact plants before treatment (T0) or grown 10 h with 100 mM mannitol (black bar) or sucrose (gray bar). Data are the means ± s.e. of n=3 replicates. Letters indicate significant differences (Ŭ= 0.05).

Supplementary Figure S7

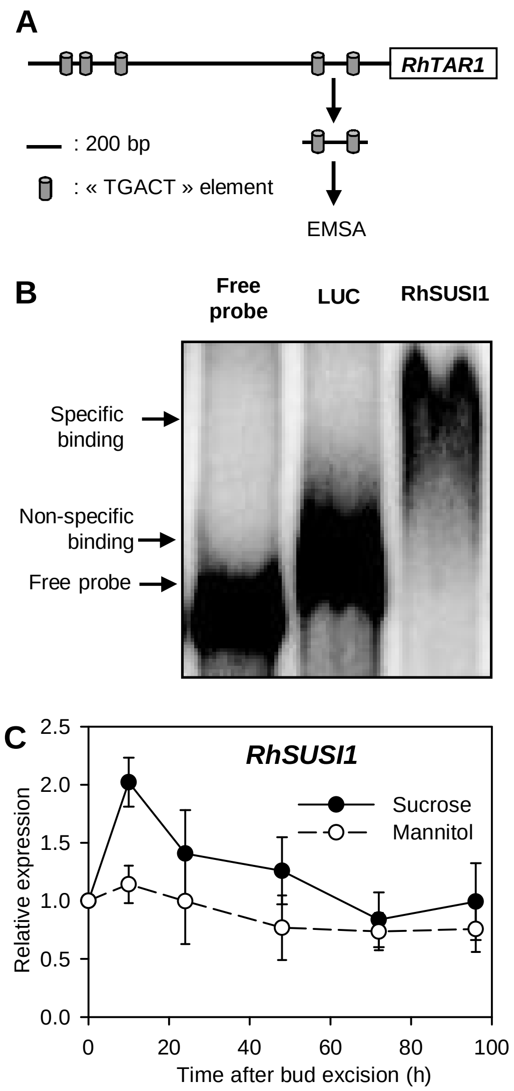

Supplementary Figure S7. (**A**) Localization of the ñTGACTò W-box elements on the promoter of *RhTAR1* and fragment used for EMSA. (**B**) EMSA performed on a native 387-bp fragment of *pRhTAR1*. Probe mobility was tested in the presence of LUCIFERASE (LUC) or RhSUSI1 and compared to a free probe. (**C**) Relative expression of *RhSUSI1* in buds grown with 100 mM mannitol or sucrose. Data are means ± s.e. of n=3 measurements on a pool of 60 buds.

Supplementary Figure S8

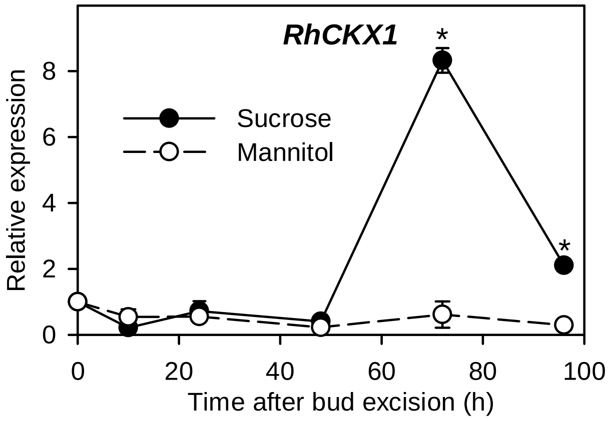

Supplementary Figure S8. Relative expression of *RhCKX1* in nodal stems grown for 96 h with 100 mM mannitol or sucrose. Data are means ± s.e. of n=3 measurements on a pool of 60 buds. Asterisks indicate significant differences between sucrose and mannitol for each time-point.

Supplementary Figure S9

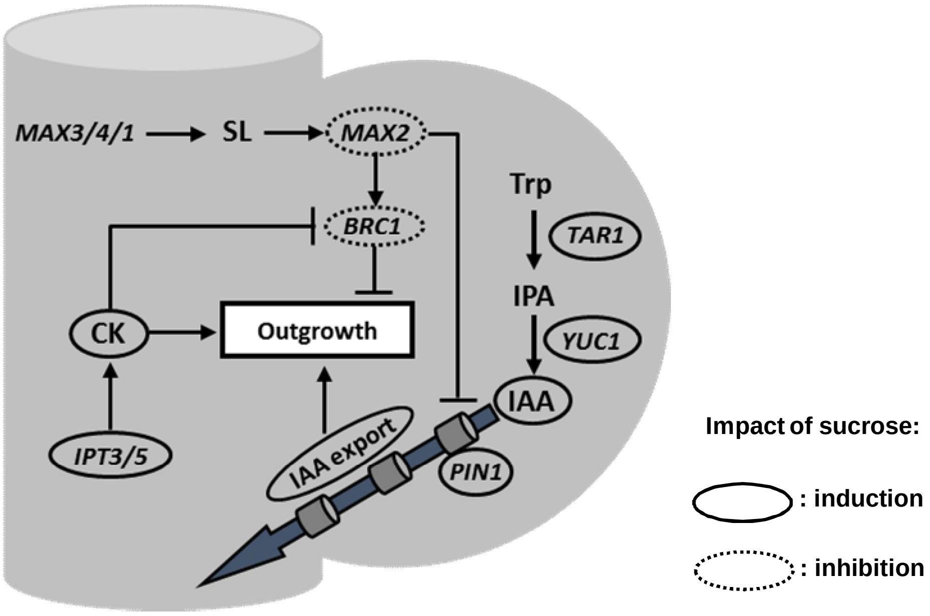

Supplementary Figure S9. Schematic representation of the impact of sucrose on the hormonal regulation during bud release. Sucrose impacts the second messenger-based model by (i) up-regulating cytokinins (CK) synthesis through the induction of *IPT3/5* and (ii) down-regulating strigolactones (SL) signaling through the inhibition of *MAX2* and *BRC1*. Sucrose impacts the auxin canalization-based model by sucrose induces (i) auxin synthesis from tryptophan (Trp) and indole-3-pyruvate (IPA) by up-regulating *TAR1* and *YUC1/2* and (ii) auxin export from bud by up-regulating *PIN1* polarization at the plasma membrane, finally leading to the establishment of an auxin transport canal between bud and stem. Ellipses in full line represent the actors up-regulated by sucrose and ellipses in dotted line represent those inhibited by sucrose.
